# c-Met in esophageal squamous cell carcinoma: an independent prognostic factor and potential therapeutic target

**DOI:** 10.1186/s12885-015-1450-3

**Published:** 2015-06-03

**Authors:** Yohei Ozawa, Yasuhiro Nakamura, Fumiyoshi Fujishima, Saulo JA Felizola, Kenichiro Takeda, Hiroshi Okamoto, Ken Ito, Hirotaka Ishida, Takuro Konno, Takashi Kamei, Go Miyata, Noriaki Ohuchi, Hironobu Sasano

**Affiliations:** 1 Division of Advanced Surgical Science and Technology, Tohoku University Graduate School of Medicine, 1-1 Seiryo-machi, Aoba-ku Sendai, 980-8574 Japan; 2 Department of Pathology, Tohoku University Graduate School of Medicine, Sendai, Japan; 3 Department of Pathology, Tohoku University Hospital, Sendai, Japan

**Keywords:** c-Met, Esophageal squamous cell carcinoma, Hepatocyte growth factor, Immunohistochemistry, Molecular targeted therapy

## Abstract

**Background:**

c-Met is widely known as a poor prognostic factor in various human malignancies. Previous studies have suggested the involvement of c-Met and/or its ligand, hepatocyte growth factor (HGF), in esophageal squamous cell carcinoma (ESCC), but the correlation between c-Met status and clinical outcome remains unclear. Furthermore, the identification of a novel molecular therapeutic target might potentially help improve the clinical outcome of ESCC patients.

**Methods:**

The expression of c-Met and HGF was immunohistochemically assessed in 104 surgically obtained tissue specimens. The correlation between c-Met/HGF expression and patients’ clinicopathological features, including survival, was evaluated. We also investigated changes in cell functions and protein expression of c-Met and its downstream signaling pathway components under treatments with HGF and/or c-Met inhibitor in ESCC cell lines.

**Results:**

Elevated expression of c-Met was significantly correlated with tumor depth and pathological stage. Patients with high c-Met expression had significantly worse survival. In addition, multivariate analysis identified the high expression of c-Met as an independent prognostic factor. Treatment with c-Met inhibitor under HGF stimulation significantly inhibited the invasive capacity of an ESCC cell line with elevated c-Met mRNA expression. Moreover, c-Met and its downstream signaling inactivation was also detected after treatment with c-Met inhibitor.

**Conclusions:**

The results of our study identified c-Met expression as an independent prognostic factor in ESCC patients and demonstrated that c-Met could be a potential molecular therapeutic target for the treatment of ESCC with elevated c-Met expression.

**Electronic supplementary material:**

The online version of this article (doi:10.1186/s12885-015-1450-3) contains supplementary material, which is available to authorized users.

## Background

Esophageal squamous cell carcinoma (ESCC) is an aggressive malignancy. Recently, several molecular markers have been identified as prognostic factors for ESCC, and the development of preoperative chemo-irradiation, surgical techniques, and postoperative chemotherapy has been reported [1]. However, the survival rate of ESCC patients remains dismal [2]. In addition, molecular targeted therapy has not been fully developed for ESCC. Therefore, the discovery of novel therapeutic targets is considered pivotal for the treatment of ESCC.

c-Met, a transmembrane receptor tyrosine kinase, is composed of α- and β-chains connected by a disulfide linkage [3]. It is activated upon the binding of hepatocyte growth factor (HGF) via auto-phosphorylation of its tyrosine kinase domain, resulting in cell motility and proliferation and possibly affecting clinical outcome [4]. Therefore, c-Met activation plays pivotal roles during embryogenesis and wound repair [3]. However, such activation also promotes tumor progression, invasion, and metastasis in cancer patients [3, 4]. The overexpression of c-Met and/or its correlation with poor prognosis has been reported in various human malignancies, including lung cancer [5, 6], breast cancer [7, 8], head and neck cancer [9–11], gastric cancer [12–14], colorectal cancer [15], bladder cancer [16], uterine cervix carcinoma [17], and esophageal adenocarcinoma [18]. In addition, the frequency of *c-Met* gene amplification and mutation in human malignancies have been reported to range from 1.4 % to 7.2 % for gene amplification [19–21] and 1.7 % to 3.3 % for mutation [19, 21] in lung cancer, 1.5 % to 10.2 % for amplification in gastric cancer [14, 22, 23], 2 % for amplification in esophagogastric adenocarcinoma [24], 13.2 % for mutation in papillary renal carcinoma [25], and 26.7 % for mutation in head and neck squamous cell carcinoma [26]. Therefore, c-Met is currently considered a potential therapeutic target molecule in various types of human malignancies [27]. Recently, the presence of *c-Met* gene amplification has been reported in ESCC [28]. However, the correlation between c-Met status and survival of ESCC patients is virtually unexplored despite the reported correlation of c-Met and/or HGF status with various clinicopathological features of ESCC [29, 30].

Therefore, in this study, we examined the clinical and biological significance of c-Met in ESCC and evaluated the potential of c-Met as a molecular therapeutic target using *in vitro* experiments.

## Methods

### Tissue samples

We examined tissue samples from 104 primary ESCC patients who underwent surgery without neoadjuvant therapy from January 2000 to December 2006 at the Tohoku University Hospital, Sendai, Japan. The final diagnosis was made based on the 6th edition of the tumor-node-metastasis classification of malignant tumors by the Union for International Cancer Control [31]. Patients diagnosed with pT1a pathological stage and/or variant tumor components were excluded from the study. The post-surgery follow-up period was at least 5 years in all patients examined in this study. Clinicopathological variables of the patients examined are summarized in Table 1. The study protocol was approved by the Ethics Committee of the Tohoku University School of Medicine (Accession No. 2012-1-213), and informed consent was obtained from all patients prior to surgery.

**Table 1:** Relationship between c-Met/HGF expression and clinicopathological features

Variable		c-Met expression		*P*-value	HGF expression		*P*-value
		Low	High		Low	High	
Age (mean = 64 years old)	<64	15	36	0.769	33	18	0.219
	≥64	17	36		28	25	
Gender	Male	27	59	0.762	53	33	0.178
	Female	5	13		8	10	
Smoking history^a^	Presence	27	56	0.802	49	34	0.987
	Absence	5	12		10	7	
Alcohol consumption history^a^	Presence	25	54	0.77	49	30	0.116
	Absence	6	11		7	10	
Tumor size^b^ (mean = 48.7 mm)	<48.7	20	38	0.357	35	23	0.694
	≥48.7	12	34		26	20	
Tumor differentiation^b^	Well	9	17	0.831	10	16	0.024^c^
	Moderate	18	45		39	24	
	Poor	5	10		12	3	
Growth pattern^b^	INFa	8	18	0.901	16	10	0.568
	INFb	18	43		37	24	
	INFc	6	11		8	9	
Lymphatic invasion^b^	ly0	8	23	0.475	22	9	0.097
	ly1–3	24	49		39	34	
Vessel invasion^b^	v0	11	15	0.141	16	10	0.73
	v1–3	21	57		45	33	
Tumor depth^d^	T1/T2	17	20	0.013^c^	27	10	0.028^c^
	T3/T4	15	52		34	33	
Lymph node metastasis^d^	N0	13	23	0.39	27	9	0.014^c^
	N1	19	49		34	34	
Distant metastasis^de^	M0	29	66	0.862	58	37	0.107
	M1	3	6		3	6	
Pathological stage^d^	I/II	23	32	0.010^c^	38	17	0.022^c^
	III/IV	9	40		23	26	
HGF expression	Low	21	40	0.336			
	High	11	32				

*HGF* hepatocyte growth factor, *INF* infiltration

^a^Data were not available for a few patients

^b^Histopathological features based on the Japanese Classification of Esophageal Cancer, 10th edition (Japan Esophageal Society 2009)

^c^ indicates statistical significance

^d^Tumor-node-metastasis (TNM) classification based on the 6th edition of the TNM classification of malignant tumors [31]

^e^All cases of distant metastasis were that of the supraclavicular lymph nodes

### Immunohistochemistry

Immunohistochemistry was performed using anti-c-Metβ polyclonal antibody (IBL, Gunma, Japan; 1:50 dilution) and anti-HGFα polyclonal antibody (IBL, Gunma, Japan; 1:100 dilution). All surgical pathology specimens, obtained from the sites of deepest invasion, were sectioned at 3-μm thickness. Antigen-retrieval was performed in 0.01 M citrate buffer (pH 6.0) by heating in a microwave. The slides were then washed with phosphate-buffered saline (PBS) and incubated with protein blocking solution (Histofine Kit; Nichirei, Tokyo, Japan) at room temperature. They were reacted with the primary antibodies overnight at 4 °C. Endogenous peroxidase activity was blocked by incubating the reacted slides in 0.3 % hydrogen peroxidase with methanol. Slides were then incubated with biotinylated goat anti-rabbit IgG (Nichirei) and horseradish peroxidase-conjugated streptavidin (Nichirei). The antigen-antibody complex was visualized with 3.3′-diaminobenzidine and counterstained with hematoxylin. Normal placenta was used as the positive control for c-Met and HGF immunoreactivity. The absorption test was performed using each antigen peptides (IBL, Gunma, Japan).

### Evaluation of immunohistochemistry

All immunostained slides were evaluated by two authors (YO and YN) without prior knowledge of any clinicopathological variables. Five different high-power fields were analyzed per slide, with each field containing more than 100 carcinoma cells. The H-score was determined using the percentage of immunopositive cells and their immunointensity. Immunointensity was evaluated and scored according to the following criteria: 0, completely negative; 1+, weakly positive; 2+, moderately positive, and 3+, markedly positive. The H-score was then calculated by multiplying the percentage of immunopositive cells to the immunointensity score (H-score ranging from 0 to 300). We also determined the optimal cut-off values using the receiver operating characteristic (ROC) curve method, which indicated that “40” was the optimal cut-off for patients’ survival outcome when analyzing c-Met and HGF immunohistochemistry results (c-Met: 40.2, HGF: 40.8). It was also close to the optimal cut-offs for tumor depth (c-Met: 42.8, HGF: 40.2) and lymph node metastasis (c-Met: 55.0, HGF: 36.2). Cases with an H-score below the cut-off value were tentatively categorized as “low expression”, whereas those with an H-score greater than the cut-off value were considered as “high expression”.

### Preoperative serum biochemical test and respiratory function test

In order to evaluate the possible influence of inflammatory processes upon c-Met and HGF expression, the preoperative levels of percent vital capacity (%VC), forced expiratory volume in one second percentage (FEV 1.0 %), C-reactive protein (CRP), the retention rate of indocyanine green 15 min after administration (ICG-R15), aspartate aminotransferase (AST), and alanine aminotransferase (ALT) were evaluated because of the high frequency of alcohol consumption and smoking history in ESCC patients.

### Cell lines and culture

Three human ESCC cell lines (KYSE150, 170, and 180) were purchased from the Health Science Research Resources Bank (Tokyo, Japan). All cell lines were authenticated by using STR analysis (BEX, Tokyo, Japan) in July 2014. The three cell lines were maintained according to the manufacturer’s instruction. All cells were cultured at 37 °C in a 5 % CO_2_ incubator.

### Pharmaceutical reagents

A small-molecule inhibitor for c-Met (PF-2341066) was purchased from Selleck Chemicals (Houston, TX, USA). PF-2341066 was reconstituted in dimethyl sulfoxide (DMSO). Recombinant human HGF (rHGF) was purchased from R&D Systems (Minneapolis, MN, USA) and reconstituted in PBS with 0.1 % bovine serum albumin.

### Quantitative real-time polymerase chain reaction

TRIzol RNA Isolation Reagents (Life Technologies, Tokyo, Japan) were used for total RNA extraction according to the manufacturer’s instruction. Reverse transcription was then performed in a thermal cycler using appropriate amounts of total RNA. The measurement of mRNA expression was performed using a Light Cycler equipment (Roche, Basel, Switzerland). The sequence of the primers used was as follows: c-Met forward: 5′-CACTTCTGAGAAATTCATCAGGCTGTGAAG-3′, reverse: 5′-AGAGGACTTCGCTGAATTGACCCATG-3′, and HGF forward: 5′-TGTGCCATTCCAAATCGTCCTGGT-3′, reverse: 5′-TCAACAAACATGACTCTCCAGTAGTTGTCT-3′. RPL13A was used as a housekeeping gene for mRNA quantification.

### Immunoblotting analysis

For protein extraction, three ESCC cell lines (KYSE150, 170, and 180) were lysed and scraped in Mammalian Protein Extraction Regent with 2 % protease inhibitor, 2 % phosphatase inhibitor, and 1 % ethylenediaminetetraacetic acid. Each protein sample was subsequently resolved in 10 % sodium dodecyl polyacrylamide gels and transferred onto nitrocellulose membranes using Amersham ECL semi-dry blotters (GE Healthcare, Tokyo, Japan). After blocking of nonspecific binding for 1.5 h in 5 % skimmed milk, the membranes were reacted with primary antibodies at 4 °C overnight. They were then washed with Tris-buffered saline and reacted with secondary antibody for 1 h. Protein expression was visualized using ChemiDoc™ XRS+ System (BIORAD, Tokyo, Japan). For quantification of protein expression by c-Met and components of its downstream signaling pathways, KYSE170 cells were cultured in fetal bovine serum (FBS)-free medium for 48 h prior to protein extraction. The quantitative determination was performed using the quantification tool of Image LabTM software (BIORAD). The primary antibodies used are summarized in Table 2.

**Table 2:** Primary antibodies used for immunoblotting analysis

Antibody	Source	Clonality (clone number)	Dilution	Provider (catalog number)
c-Met β	Rabbit	P	×200	IBL, Japan (18321)
Phospho-Met (pY1234/35)	Rabbit	P	×500	CST, USA (#3126)
Akt (Pan)	Rabbit	M (11E7)	×1000	CST (#4685)
Phospho-Akt (Ser473)	Rabbit	P	×500	CST (#4060)
p44/42 MAPK (Erk1/2)	Rabbit	M (137 F5)	×1000	CST (#4695)
Phospho-P44/42 MAPK (Erk1/2) (Thr202/Tyr204)	Rabbit	M (20G11)	×500	CST (#4376)

*P* Polyclonal, *M* Monoclonal, *CST* Cell Signaling Technology, *MAPK* mitogen-activated protein kinase

### Invasion assay

The invasive properties of ESCC cells were determined using Cell Culture Inserts with transparent PET membrane (24-well, 8.0-μm pore size) and their companion plates (BD Falcon, San Jose, CA, USA). After culturing for 24 h in an FBS-free medium, three ESCC cell lines (KYSE150, 170, and 180) were seeded in the upper chambers (3 × 10^5^ cells/chamber for KYSE170 and 1 × 10^5^ cells/chamber for KYSE 150 and 180) with PF-2341066 treatment or vehicle control. These upper chambers were inserted in to a 24-well lower chamber containing FBS-free medium supplemented with 50 ng/ml of HGF or vehicle control. After the plate was cultured for 24 h at 37 °C with 5 % CO_2_, each upper chamber filter was fixed with methanol and counterstained with hematoxylin. The number of invasive tumor cells for each filter membrane was then counted in high-power fields.

### Cell proliferation assay

ESCC cells were counted using the Cell Counting Kit-8 (Dojindo, Kumamoto, Japan). Three ESCC cell lines (KYSE150, 170, and 180) were seeded in a 96-well plate (1 × 10^4^ cells/well). After the plate was cultured for 24 h in FBS-free medium, the medium was switched to one with or without 50 ng/ml of HGF and PF-2341066. Then, WST-8, a highly water-soluble tetrazolium salt, was added to each well at 24 h after PF-2341066 treatment, and absorbance at 450 nm was measured using a microplate reader (BIORAD). Equivalent volumes of DMSO were used as vehicle controls.

### Statistical analysis

Pearson’s chi-square test was used to assess the correlation of immunohistochemical protein expression with various clinicopathological features. *P-*values of <0.05 were considered statistically significant. Survival curves were generated using the Kaplan-Meier method, and differences were compared using the log-rank test. Univariate and multivariate analyses were performed using Cox’s proportional hazards model. The multivariate analysis performed in this study included clinicopathological features with a *P*-value of <0.05 in the antecedent univariate analysis, c-Met expression, and HGF expression. Student’s *t*-test was used for the analysis of preoperative biochemical values of blood samples, preoperative respiratory function test, proliferation assay, invasion assay, and immunoblotting quantification. All statistical analyses were performed using JMP Pro 9 software (SAS Institute, Cary, CA, USA).

## Results

### c-Met and HGF expression in ESCC tissue specimens

**Fig. 1:**
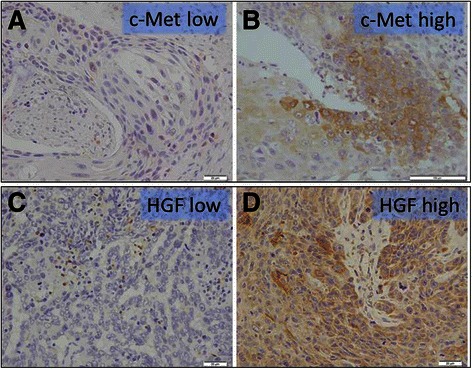
Representative microscopic images of low and high c-Met and HGF immunohistochemical staining. **a** c-Met low expression; the representative case shown was completely negative for c-Met immunoreactivity. **b** c-Met high expression; the representative case demonstrated c-Met immunoreactivity in the cytoplasm of carcinoma cells. **c** HGF low expression; the representative case shown was completely negative for HGF immunoreactivity. **d** HGF high expression; the representative case demonstrated HGF immunoreactivity in the cytoplasm of carcinoma cells. HGF, hepatocyte growth factor

Figure 1 shows representative microscopic images of c-Met and HGF immunostaining. c-Met high expression was detected in 69.2 % (72/104) of the patients examined. c-Met immunoreactivity was mostly detected in the cytoplasm of cancer cells (Fig. 1b). HGF immunoreactivity was mainly present in the cytoplasm of carcinoma cells (Fig. 1d) and intratumoral stromal cells, including cancer-associated fibroblasts (CAF). HGF immunoreactivity of intratumoral stromal cells was heterogeneous and difficult to evaluate. Therefore, we only considered cytoplasmic immunoreactivity in carcinoma cells for statistical analysis.

### The association between c-Met/HGF status and patients’ clinicopathological features

c-Met status was significantly correlated with tumor depth (*P* = 0.013) and pathological stage (*P* = 0.010). On the other hand, HGF status was significantly correlated with tumor differentiation (*P* = 0.024), tumor depth (*P* = 0.028), lymph node metastasis (*P* = 0.014), and pathological stage (*P* = 0.022). However, no statistically significant correlation was detected between c-Met and HGF status of the patients. Results of the correlation analysis of ESCC patients’ clinicopathological variables and c-Met/HGF immunoreactivity in carcinoma cells are summarized in Table 1.

### The association between c-Met/HGF high expression and patient survival

**Fig. 2:**
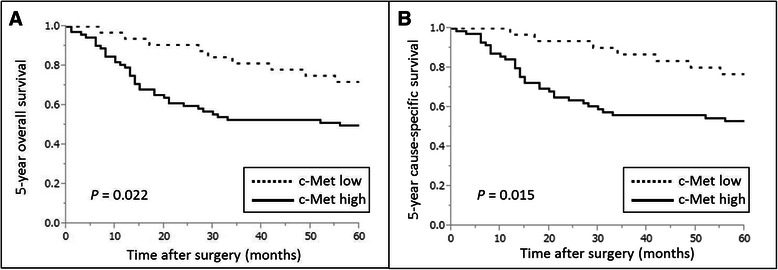
Kaplan-Meier curves according to c-Met expression. **a** The 5-year overall survival of ESCC patients with high c-Met expression was significantly worse than that of patients with low c-Met expression. **b** The differences in cause-specific survival between the two groups of ESCC patients were even more statistically significant than those observed in their 5-year overall survival. ESCC, esophageal squamous cell carcinoma

The 5-year overall survival rate of patients with c-Met high expression was significantly lower than that of those in the c-Met low expression group (*P* = 0.022) (Fig. 2a). This difference was even more pronounced in cause-specific survival (CSS) (*P* = 0.015) (Fig. 2b). Univariate analysis revealed that patient survival was significantly associated with sex (*P* = 0.036), lymphatic invasion (*P* = 0.015), tumor depth (*P* = 0.015), lymph node metastasis (*P* < 0.001), pathological stage (*P* < 0.001), and c-Met expression (*P* = 0.017) (Table 3). In addition, multivariate analysis identified high expression of c-Met (*P* = 0.033) and lymph node metastasis (*P* = 0.025) as independent prognostic factors (Table 4). However, no significant differences in 5-year overall survival and CSS between patients with high and low HGF expression were observed. Patients with high HGF expression tended to have a lower survival rate (Additional file 1), but the difference did not reach statistical significance. Furthermore, patients with high c-Met and HGF demonstrated worse clinical outcomes than others, but the 5-year overall survival of those with such a combined status of c-Met and HGF was not significantly different from that of others (Additional file 2).

**Table 3:** Univariate analysis of patients’ 5-year overall survival

Variable	*P*-value	Hazard ratio (95 % CI)
Age (≥64/<64 years) (mean, 64 years)	0.992	1.003 (0.557–1.809)
Sex (male/female)	0.036^a^	2.621 (1.057–8.724)
Tumor size (≥48.7/<48.7 mm) (mean, 48.7 mm)	0.099	1.639 (0.912–2.971)
Tumor differentiation (well/moderate)	0.302	0.682 (0.303–1.390)
(well/poor)	0.143	0.484 (0.185–1.290)
(moderate/poor)	0.41	0.710 (0.340–1.671)
Growth pattern (INFa/INFb)	0.725	0.878 (0.402–1.774)
(INFa/INFc)	0.158	0.528 (0.216–1.290)
(INFb/INFc)	0.193	0.602 (0.297–1.316)
Lymphatic invasion (ly1–3/ly0)	0.015*	2.392 (1.174–5.536)
Venous invasion (v1–3/v0)	0.763	1.109 (0.581–2.295)
Tumor depth (pT3–4/pT1–2)	0.015^a^	2.265 (1.164–4.835)
Lymph node metastasis (pN1/pN0)	<0.001^a^	4.692 (2.139–12.358)
Distant metastasis (pM1/pM0)	0.086	2.308 (0.875–5.069)
Pathological stage (pStage III–IV/I–II)	<0.001^a^	3.403 (1.840–6.620)
c-Met expression (high/low)	0.017^a^	2.291 (1.153–5.073)
HGF expression (high/low)	0.194	1.477 (0.817–2.662)

*CI* confidence interval, *HGF* hepatocyte growth factor, *INF* infiltration

^a^ indicates statistical significance

**Table 4:** Multivariate analysis of patients’ 5-year overall survival

Variable	*P*-value	Relative risk (95 % CI)
Sex (male/female)	0.193	1.944 (0.737–6.710)
Lymphatic invasion (ly1–3/ly0)	0.222	1.628 (0.756–3.925)
Tumor depth (pT3–4/pT1–2)	0.26	1.911 (0.644–7.047)
Lymph node metastasis (pN1/pN0)	0.025^a^	3.852 (1.169–16.335)
Pathological stage (pStage III–IV/I–II)	0.814	0.856 (0.215–2.898)
c-Met expression (high/low)	0.033^a^	2.237 (1.066–5.190)
HGF expression (high/low)	0.821	1.075 (0.572–2.012)

*CI* confidence interval, *HGF* hepatocyte growth factor

^a^indicates statistical significance

### The association between c-Met/HGF expression and preoperative serum biochemical test and respiratory function test

There were no significant correlations between c-Met/HGF expression and preoperative AST, ALT, ICG-R15, %VC and FEV 1.0 % in ESCC patients (data not shown).

### c-Met and HGF expression in ESCC cell lines

**Fig. 3:**
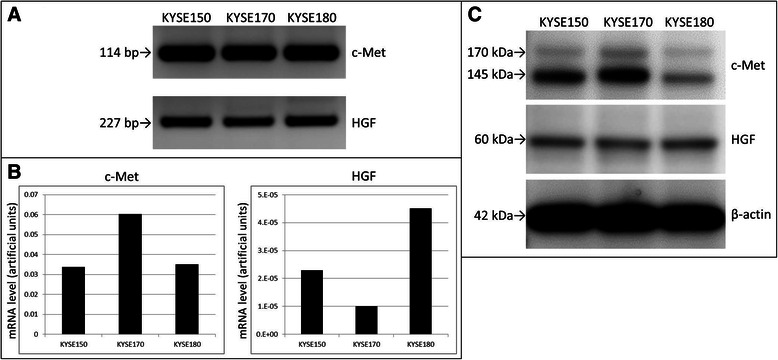
mRNA and protein levels of c-Met and HGF in ESCC cell lines. **a** Electrophoretic analysis of the polymerase chain reaction products showed each bands as expected for c-Met and HGF. **b** Quantitative analysis revealed the highest c-Met level in KYSE170 and very small amounts of HGF mRNA in all tested lines. **c** Western blot analysis confirmed the expression of c-Met and HGF proteins in all three cell lines. HGF, hepatocyte growth factor; ESCC, esophageal squamous cell carcinoma

c-Met and HGF mRNA was detectable in all three cell lines examined (Fig. 3a). The amounts of HGF mRNA were much lower than those of c-Met (Fig. 3b). We also confirmed the expression of c-Met and HGF proteins in all three cell lines using immunoblotting (Fig. 3c). Of these, KYSE170 had the highest amounts of c-Met mRNA.

### The invasion capacity of ESCC cells was promoted by rHGF stimulation but inhibited by PF-2341066

**Fig. 4:**
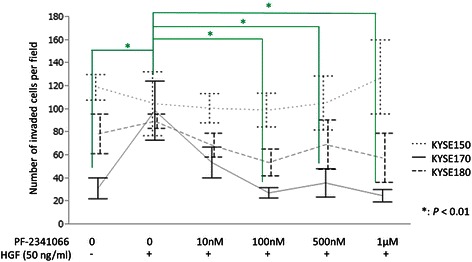
Variation in invasive capacity of three ESCC cell lines. In KYSE170 cells, the invasion capacity of cells was markedly increased by rHGF pre-stimulation and decreased by PF-2341066. KYSE 180 cells demonstrated similar invasive tendencies as those observed in KYSE 170 cells, but the changes were not statistically significant. However, the invasion capacity of KYSE 150 cells did not differ significantly under rHGF pre-stimulation and/or PF-2341066 treatment. Green statistical comparison bars and stars represent the KYSE170 cell line. rHGF, recombinant hepatocyte growth factor

The invasiveness of KYSE170 cells was promoted by rHGF. When both rHGF and PF-2341066 were absent, the number of invaded cells in invasion assay was 31.6 ± 9.0, but that increased to 99.0 ± 25.6 when only rHGF was added (*P* = 0.0019). However, the number of invaded cells decreased under PF-2341066 treatment to 54 ± 13.3 at 10 nM, 27.6 ± 4.5 at 100 nM, 36.2 ± 12.3 at 500 nM, and 25.2 ± 5.4 at 1 μM. In addition, treatment with 100 nM, 500 nM, and 1 μM of PF-2341066 significantly decreased cell invasiveness (*P* = 0.001, 0.003, and <0.001) (Fig. 4). KYSE 180 cells demonstrated similar invasive tendencies as those observed in KYSE 170 cells, but the changes were not statistically significant. However, the invasion capacity of KYSE 150 cells did not differ significantly under rHGF pre-stimulation and/or PF-2341066 treatment.

### Inhibition of ESCC cell proliferation by PF-2341066

**Fig. 5:**
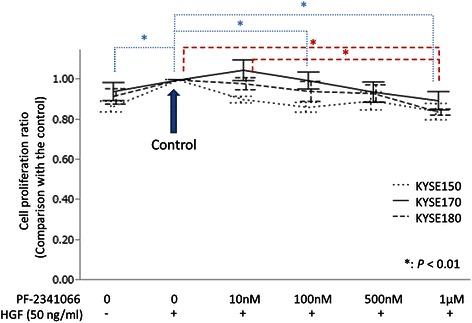
Variation in cell proliferation of three ESCC cell lines. In all three cell lines examined, the variations of cell proliferation increased by rHGF pre-stimulation and decreased by PF-2341066 were only limited to about less than 15 %. Blue and red statistical comparison bars and stars represent the KYSE150 and KYSE180 cell lines, respectively

The proliferation of the three ESCC cell lines (KYSE150, 170 and 180) under PF-2341066 treatment was evaluated as a decrease percentage compared to control (rHGF+/PF-2341066-). When 1 μM of PF-2341066 was added, the proliferation of KYSE150 cells decreased 16 % (*P* = 0.001); that of KYSE170 decreased 11 % (*P* = 0.11), and KYSE180 cell proliferation decreased 16 % (*P* = 0.003) (Fig. 5). However, when the proliferation was compared between untreated cells and those receiving rHGF but not PF-2341066 (control), incremental cell proliferation was observed in all three cell lines, but the differences did not reach statistical significance except for KYSE150.

### Activation of c-Met and its downstream signaling pathways affected by PF-2341066

**Fig. 6:**
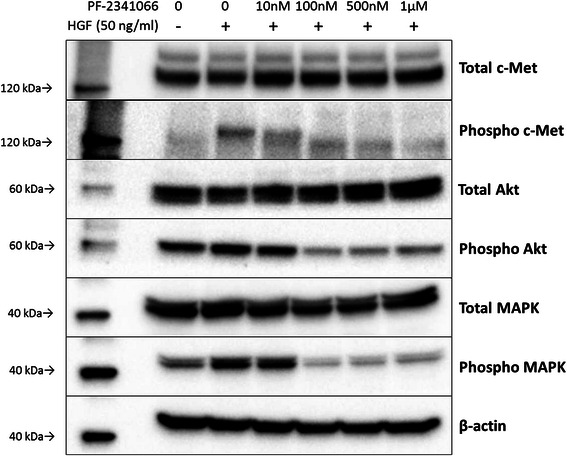
Western blot analysis of KYSE170 cells. Immunoblot analysis revealed that phospho-c-Met protein expression was markedly increased by rHGF pre-stimulation and decreased by PF-2341066 treatment. Similarly, phospho-Akt and phosphor-MAPK protein expression was also increased by rHGF pre-stimulation and decreased by PF-2341066. rHGF, recombinant hepatocyte growth factor; MAPK, mitogen-activated protein kinase

**Fig. 7:**
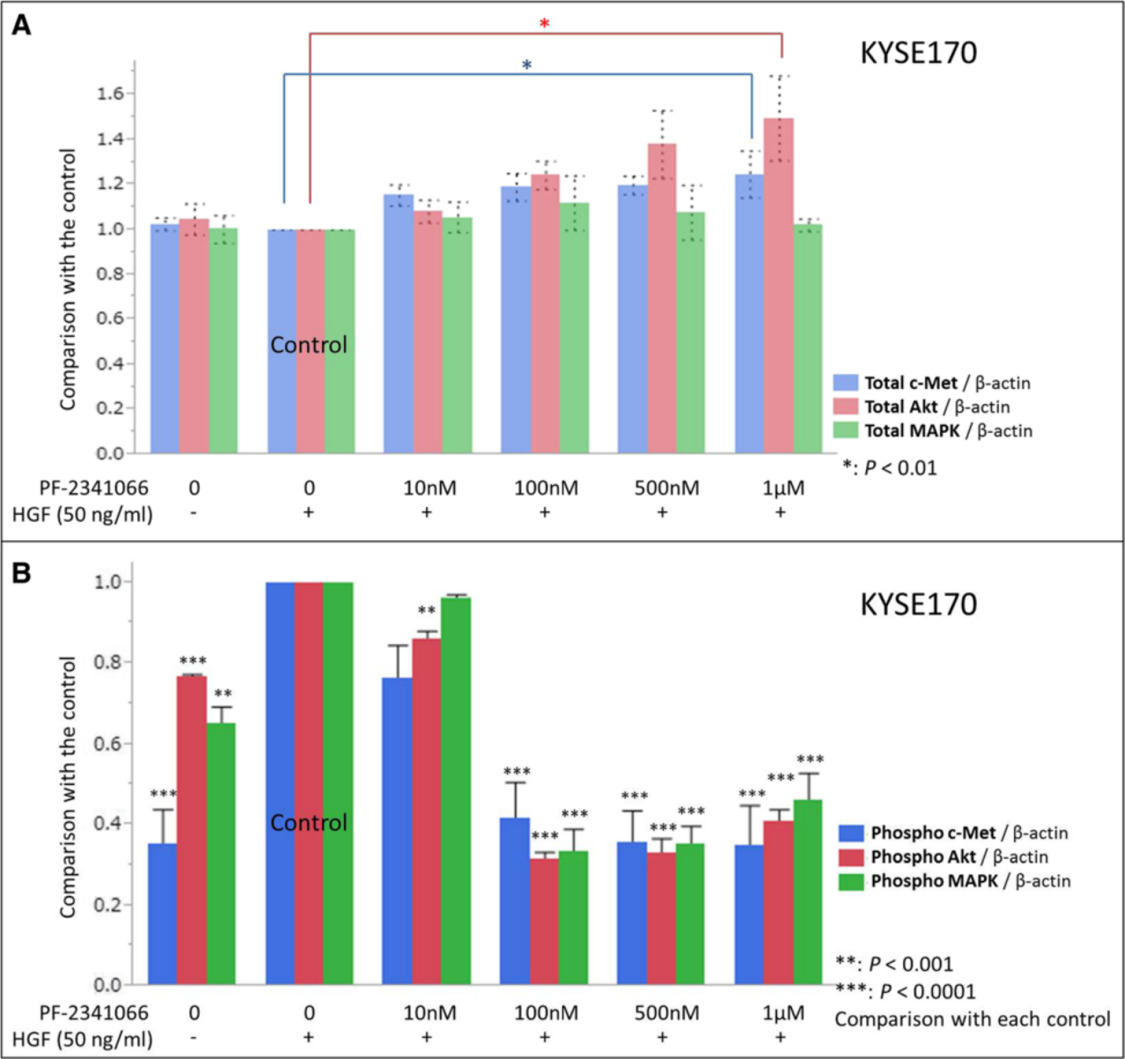
Quantitative analysis of KYSE170 cells’ immunoblot. The phosphorylated forms of c-Met, Akt, and MAPK were significantly increased by rHGF pre-stimulation and decreased under PF-2341066 treatment. However, the protein levels of total c-Met, Akt, and MAPK were not affected by such treatments

The activation of c-Met and its downstream signaling pathway was evaluated via immunoblotting analysis of KYSE170 cells, which demonstrated marked inhibitory effects in the invasion assay. In the absence of rHGF, total c-Met protein and only weak phospho-c-Met expression were detected. In contrast, the expression of phospho-MAPK and phospho-Akt was detected without rHGF. However, when rHGF was added, phosphor-c-Met was clearly detected, and the immunoreactivity of both phospho-MAPK and phospho-Akt was more prominent than that in the absence of rHGF (Fig. 6). Expression of the three phosphorylated proteins was remarkably inhibited by PF-2341066 (Fig. 6). Quantitative analysis of immunoblotting results demonstrated that phospho-c-Met expression significantly decreased in a concentration-dependent manner under PF-2341066 treatment (23.7 % decrease at 10 nM; 58.6 % at 100 nM, *P* < 0.0001; 64.6 % at 500 nM, *P* < 0.0001; and 65.3 % at 1 μM, *P* < 0.0001) (Fig. 7). In addition, phospho-Akt expression significantly decreased to 14.0 % at 10 nM, 68.5 % at 100 nM, 67.1 % at 500 nM, and 59.3 % at 1 μM, whereas that of phospho-MAPK significantly decreased to 66.5 % at 100 nM, 65.0 % at 500 nM, and 54.1 % at 1 μM (Fig. 7).

## Discussion

The overexpression of c-Met has been reported in ESCC [32], but its biological and clinical significance remains virtually unknown [33], despite the reported roles of paracrine signaling from CAF, involving HGF, in ESCC invasion by Grugan *et al.* [32]. In addition, elevated serum or tissue HGF levels have been reported to be associated with adverse clinical outcome in ESCC patients [34, 35]. The results of our present study indicated that elevated expression of c-Met was significantly correlated with tumor depth and pathological stage of ESCC patients. In addition, increased HGF expression was significantly correlated with tumor differentiation, tumor depth, lymph node metastasis, and pathological stage. However, these results might be challenging to analyze because the cut-offs were determined via patients’ survival. Our survival analysis revealed that patients with high c-Met expression had significantly worse 5-year overall survival and CSS than those without. Furthermore, the statistical difference of CSS between the two groups was more prominent than that of 5-year overall survival. This result suggested that c-Met high expression could be a prognostic factor in ESCC patients. In fact, c-Met status of carcinoma cells was identified as an independent prognostic factor for clinical outcomes in these patients. Although c-Met overexpression is thought to promote tumor progression and/or serve as an independently poor prognostic factor in various carcinomas [5–18], to the best of our knowledge, this study was the first investigation to report its clinical significance in ESCC. Our results demonstrated that c-Met overexpression could promote cell growth and invasion in ESCC.

However, the correlation between c-Met and HGF expression did not reach statistical significance. This may be explained by the fact that HGF is secreted by carcinoma cells as well as CAFs [32] and in other inflammatory processes, including alcoholic hepatitis, cirrhosis, smoking, and chronic obstructive pulmonary disease [36, 37]. We therefore analyzed the correlation between c-Met/HGF immunoreactivity and preoperative levels of %VC, FEV 1.0 %, CRP, ICG-R15, AST, and ALT owing to the high frequency of alcohol consumption and smoking history in ESCC patients, but found no significant correlation. Nonetheless, HGF immunoreactivity in CAFs was heterogeneous and difficult to evaluate. Therefore, functional HGF to activate c-Met was reasonably postulated to be secreted from not only CAFs and other inflammatory processes but also tumor cells, although further investigations for clarification is needed.

In this study, we also confirmed mRNA and protein expression of c-Met and HGF in three ESCC cell lines. Saeki *et al.* [33] previously reported c-Met mRNA and protein expression in six different ESCC cell lines. Hu *et al.* [29] also described the overexpression of c-Met mRNA and protein in ESCC compared to normal epithelium. In our study, KYSE170 cells showed the highest expression of c-Met mRNA. On the other hand, mRNA and protein levels of HGF in ESCC cell lines are unknown because the HGF secretory mechanism has been postulated to be derived via paracrine signaling from CAFs [32, 34]. However, in this study, we did detect HGF expression in all three ESCC cell lines studied albeit low levels.

The results of our *in vitro* studies demonstrated the important role of HGF as a c-Met activator and the efficacy of the c-Met small-molecule inhibitor PF-2341066, especially in KYSE170 cells with the highest c-Met mRNA expression among the three ESCC cell lines tested. The invasive potential of KYSE170 cells was significantly increased by rHGF pre-stimulation and decreased by PE-2341066 treatment. In addition, the changes in phospho-c-Met protein levels were also highly HGF dependent. However, the inhibitory effects of ESCC cell proliferation by PF-2341066 were only marginally detected in the three cell lines examined in our study. These results indicated that the inhibitory effects of PF-2341066 in ESCC were detected only in c-Met high expression cells, and the drug mainly suppressed cell invasive potential rather than cell proliferation. c-Met activation is well known to occur via homodimerization and autophosphorylation upon binding to its specific ligand, HGF, under physiological conditions [3]. However, in cancer, c-Met was not only activated by the above-mentioned HGF-dependent paracrine and autocrine mechanisms but also via other HGF-dependent or independent fashions such as in cases of c-Met gene amplification or mutations [28]. The results of our study demonstrated that c-Met was activated by HGF stimulation in ESCC, as in other malignancies. Although Kato *et al.* reported approximately 1 % (2/196) prevalence of c-Met gene amplification in ESCC patients [27], the status of *c-Met* gene mutation or amplification in ESCC is virtually unknown.

The important downstream signaling of c-Met includes the MAPK cascade and PI3K/Akt signaling pathway, which mainly regulates cell proliferation and motility [3]. We showed that ESCC cell invasive property was significantly inhibited by PF-2341066 in the presence of rHGF in one of the three ESCC cell lines examined. In addition, we also demonstrated that the expression levels of phosphorylated c-Met, MAPK, and Akt proteins were all significantly down regulated by treatment with PF-2341066. Marked inhibition of MAPK and Akt signaling were considered important. Zillhardt *et al.* reported the inhibition of MAPK and Akt signaling by PF-2341066 in ovarian cancer cells [38], which was consistent with our results. Knowles *et al.* reported the inhibition of Akt signaling but not of the MAPK cascade by PF-2341066 in head and neck squamous cell carcinoma cells, possibly due to the high basal level of phosphorylated MAPK in these cells prior to HGF stimulation [39]. Our results indicated that the motility of ESCC cells, at least in those with high c-Met expression, was regulated by c-Met activation upon HGF stimulation, and the process was mediated by both MAPK and Akt signaling pathways. Therefore, c-Met could be a potential molecular therapeutic target for the treatment of ESCC with high c-Met expression as in other human malignancies [18, 38–41]. However, c-Met regulates many downstream signaling mediators, including not only MAPK and Akt but also a signal transducer and activator. Therefore, further investigations are needed to elucidate c-Met functional behavior in ESCC.

PF-2341066 is also known to inhibit the protein activation processes resulted from gene rearrangement of anaplastic lymphoma kinase and ROS1 tyrosine kinase [42, 43] in addition to c-Met inhibition. However, to the best of our knowledge, such rearrangements have not been reported in ESCC. Additionally, our results demonstrated that the inhibition of cell function by PF-2341066 depended on the presence of HGF, a c-Met specific ligand. Thus, PF-2341066 functioned as an efficient c-Met inhibitor in the context of our study.

## Conclusion

In conclusion, the results of our study identified c-Met expression in carcinoma cells as an independent prognostic factor for ESCC and demonstrated the potential of c-Met as a molecular therapeutic target for the treatment of high c-Met expressing ESCC. However, further genetic and/or biological investigations are warranted.

## Additional files

Additional file 1: **Patient survival according to HGF expression. No statistically significant differences in 5-year overall survival (A) or cause-specific survival (B) according to HGF expression were observed via the log-rank test.** However, patients with high HGF expression tended to have a lower survival rate. HGF, hepatocyte growth factor.
Additional file 2: **Patient survival according to the combination expression of c-Met and HGF.** Patients with high c-Met and HGF demonstrated adverse clinical outcomes compared to others, but the 5-year overall survival of those with a combination of high c-Met and HGF was not significantly different to that of other patient groups. HGF, hepatocyte growth factor.

## Abbreviations

ESCC: Esophageal squamous cell carcinoma
HGF: Hepatocyte growth factor
PBS: Phosphate buffered saline
FBS: Fetal bovine serum
DMSO: Dimethyl sulfoxide
rHGF: Recombinant human hepatocyte growth factor
CSS: Cause-specific survival
MAPK: Mitogen-activated protein kinase
CAF: Cancer-associated fibroblasts
