# Supplementary figures and images for: c-Met in esophageal squamous cell carcinoma: an independent prognostic factor and potential therapeutic target
Source: BMC Cancer. 2015 Jun 3;15:451. doi: 10.1186/s12885-015-1450-3 (PMC4453225; doi:10.1186/s12885-015-1450-3)

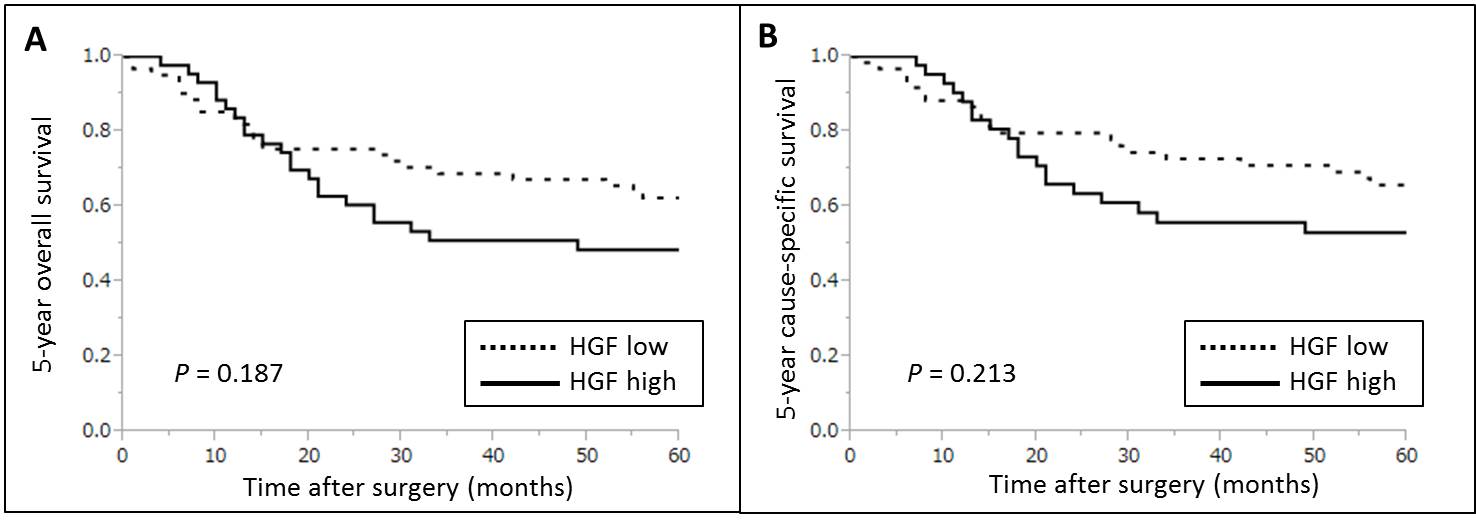

Supplement: Additional file 1: — Patient survival according to HGF expression. No statistically significant differences in 5-year overall survival (A) or cause-specific survival (B) according to HGF expression were observed via the log-rank test. However, patients with high HGF expression tended to have a lower survival rate. HGF, hepatocyte growth factor. [file 12885_2015_1450_MOESM1_ESM.jpeg]

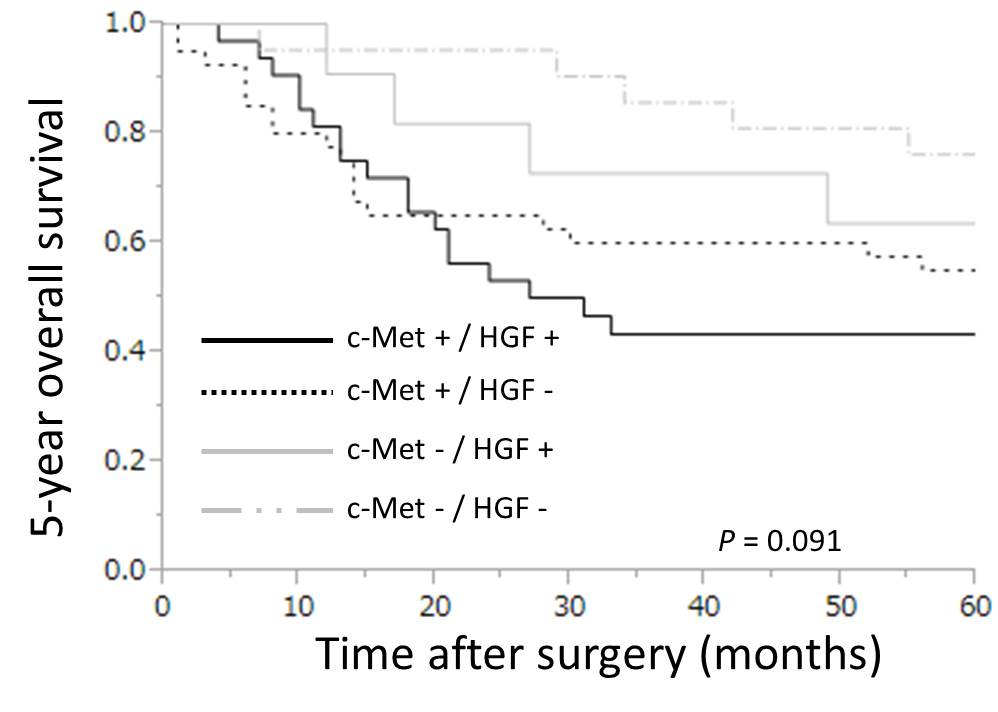

Supplement: Additional file 2: — Patient survival according to the combination expression of c-Met and HGF. Patients with high c-Met and HGF demonstrated adverse clinical outcomes compared to others, but the 5-year overall survival of those with a combination of high c-Met and HGF was not significantly different to that of other patient groups. HGF, hepatocyte growth factor. [file 12885_2015_1450_MOESM2_ESM.jpeg]
